# A VSV-vector vaccine simultaneously targeting H5N1 hemagglutinin and matrix protein 2 induces robust neutralizing and ADCC antibody responses and provides full protection against lethal H5N1 infection in a mouse model

**DOI:** 10.1128/jvi.00097-26

**Published:** 2026-06-16

**Authors:** Zhujun Ao, Robert Vendramelli, Maryann Buyu, Thang Truong, Dayu Liu, Nick Gao, Heqing Ma, Stephen Amos, Peter Lawrynuik, Maurice Nyamekye, Keith R. Fowke, Peter Pelka, Maya Shmulevitz, Sam K. P. Kung, Darwyn Kobasa, Xiaojian Yao

**Affiliations:** 1 Laboratory of Molecular Human Retrovirology, University of Manitoba 8664, Winnipeg, Manitoba, Canada; 2 Department of Medical Microbiology, University of Manitoba 8664, Winnipeg, Manitoba, Canada; 3 Special Pathogens Program, National Microbiology Laboratory, Public Health Agency of Canada 85072 , Winnipeg, Manitoba, Canada; 4 Department of Immunology, Max Rady College of Medicine, Rady Faculty of Health Sciences, University of Manitoba 12359, Winnipeg, Manitoba, Canada; 5 Department of Microbiology, University of Manitoba 468335, Winnipeg, Manitoba, Canada; 6 Department of Medical Microbiology and Immunology, Li Ka Shing Institute for Virology, University of Alberta 839763, Edmonton, Alberta, Canada; University of Kentucky College of Medicine, Lexington, Kentucky, USA

**Keywords:** multivalent influenza vaccine, VSV vector, avian influenza, H5N1, hemagglutinin, M2 ectodomain, neutralizing antibody, ADCC activity, intranasal vaccination

## Abstract

Avian influenza A viruses, especially highly pathogenic avian influenza (HPAI) viruses, pose a significant public health threat, and a multivalent vaccine is the primary prophylactic measure to control these viruses. To establish such a vaccine, we generated two multivalent vesicular stomatitis virus (VSV)-based vaccine candidates that simultaneously target H5N1 hemagglutinin (H5_05_ or H5_22_) and the ectodomain of matrix protein 2 (M2e) and characterized their ability to induce protective immune responses. Our results revealed that vaccine immunization induced high humoral immune responses against both the HPAI hemagglutinin (HA) protein and the M2 protein in mice. Intriguingly, vaccine-immunized mouse sera exhibited highly efficient neutralizing activity against the corresponding H5 pseudovirus and mediated potent and broad antibody-dependent cellular cytotoxicity (ADCC) activity against M2e derived from human and avian influenza H5, H1, H3, and H7 viruses. Furthermore, both intranasal (IN) and intramuscular immunization (IM) of V-EtM2e/H5_22_ provided efficient protection against HPAI H5N1 virus challenge in mice, with a 100% survival rate and a nondetectable viral load in several tissues. Notably, noninvasive mucosal IN delivery achieved protection equal to that of IM delivery at a 100-fold lower immunizing dose. These findings provide strong evidence for the effectiveness of a multivalent VSV-based vaccine against human (avian) influenza A viruses.

**IMPORTANCE:**

Avian influenza, especially highly pathogenic avian influenza (HPAI) viruses, can cause a highly contagious airborne disease that poses a significant public health threat. The development of a multivalent vaccine is very important to control these viral infections. In this study, we have developed a multivalent vaccine that simultaneously targets two critical HPAI H5N1 surface viral proteins and fully protects against highly pathogenic H5N1 infection in a mouse model. This study provides convincing evidence for the effectiveness of a multivalent vaccine against highly pathogenic influenza A viruses.

## INTRODUCTION

Influenza is a highly contagious airborne disease that primarily affects the respiratory tract, causing recurrent seasonal epidemics and, at times, catastrophic pandemics. Among influenza A viruses, avian influenza viruses (AIVs) are particularly concerning because of their extensive circulation in domestic and wild bird populations and their ability, under certain conditions, to cross the species barrier into mammals, including humans (1, 2). Avian influenza viruses are categorized as either low pathogenic avian influenza (LPAI) or highly pathogenic avian influenza (HPAI) viruses. LPAI viruses often cause mild or asymptomatic infections in poultry (2), whereas HPAI viruses induce severe disease with high mortality (2, 3), especially for H5N1, an HPAI subtype, identified in Guangdong, China, in 1996 as A/goose/Guangdong/1/96 (4). H5N1 has rapidly spread across Asia, Europe, the Middle East, Africa, and even North America (5, 6). Large outbreaks in migratory and wild birds have established long-term viral reservoirs, increasing the likelihood of extension to agricultural systems and human populations. In 2024, H5N1 caused a multistate *outbreak* in poultry and dairy cows in the United States, resulting in human infections primarily affecting those with occupational exposure to infected animals (7). Although sustained human-to-human transmission of this virus has not been documented, viral evolution through antigenic drift or reassortment with seasonal influenza A viruses could confer this property, making H5N1 a leading candidate for causing a pandemic. Therefore, developing vaccines for HPAI viruses is a high public health priority to combat a potential epidemic or HPAI pandemic.

Despite this urgency, human HPAI vaccine development remains in its early stages. Current influenza vaccines, which largely rely on hemagglutinin (HA) (8) or neuraminidase (NA) (9) antigens, provide strain-specific neutralizing protection and are not universal (10–12), leaving individuals vulnerable to new or divergent AIV subtypes. Furthermore, the traditional egg-based manufacturing process is unsuitable for HPAI vaccine production because of the virus’s high pathogenicity in eggs, which adds barriers to scale and costs (13). Thus, two central challenges in HPAI vaccine design are improving multivalent efficacy across diverse viral strains and reducing production costs to enable widespread access. Addressing these challenges requires novel approaches that target conserved viral components, with the ultimate goal of developing universal influenza vaccines capable of eliciting broad, durable, and affordable protection against a wide spectrum (14, 15) of influenza A viruses.

In addition to antigen design, the choice of vector plays a pivotal role in vaccine efficacy. The recombinant vesicular stomatitis virus (rVSV) vector has several advantages, including highly efficient delivery of antigens to host cells and the induction of strong humoral and cellular immune responses. Its broad host range, capacity for robust replication, and ability to be engineered for safety make rVSV an attractive platform for vaccine development. Importantly, rVSV-based vaccines have demonstrated the ability to induce durable protective immunity and can be adapted for both intramuscular and mucosal delivery, thereby supporting their versatility for controlling emerging viral threats.

Our group previously developed a recombinant vesicular stomatitis virus (rVSV)-based universal influenza vaccine candidate incorporating four tandem repeats of the influenza M2 ectodomain (tetra-M2e, tM2e) fused to the dendritic cell-targeting domain of Ebola virus glycoprotein (EΔM) (rVSV-EboΔM-tM2e) (16). This design aimed to leverage the high sequence conservation of M2e to induce antibody-mediated cellular events such as antibody-dependent cellular cytotoxicity (ADCC) (17), antibody-dependent cellular phagocytosis (ADCP) (18), and complement-dependent cytotoxicity (CDC) (19), thereby broadening protection against diverse influenza A subtypes. Our results demonstrated that the rVSV-EΔM-tM2e vaccine induced rapid and potent M2 antibody production and ADCC activity and protected mice from H1N1 and H3N2 influenza challenges. In this study, we sought to develop a VSV-based multivalent vaccine designed to simultaneously target two distinct influenza membrane antigens, the HA from a recently isolated HPAI H5N1 strain (Influenza A/Red-Tailed Hawk/ON/FAV-0473-4-2022, H5_22_) or from a 2005 H5N1 strain (Influenza A/Hanoi/30408/2005, H5_05_), and four copies of the ectodomain of influenza M2 (human/bird/swine) that fused with an Ebola virus glycoprotein dendritic cell-targeting domain (EΔM). Influenza HA is the primary target of neutralizing antibodies, yet its high variability limits cross-protection. By combining HA with the highly conserved M2e antigen, this vaccine aims to elicit both potent neutralizing antibody responses against HA and antibodies that mediate ADCC against highly conserved regions of the influenza M2 ectodomain to provide broad protection against HPAI viruses. The use of the VSV vector and EΔM for dendritic cell targeting further enhances antigen presentation and immune priming. Collectively, this approach integrates antigenic breadth with potent immune activation, offering a promising strategy toward a more universal influenza vaccine.

## MATERIALS AND METHODS

### Plasmid constructs

The cDNA encoding the EΔM-tM2e fusion was previously described (16, 20). To generate the rVSVΔG vector co-expressing both the codon-optimized genes encoding avian influenza H5N1 hemagglutinin (HA) and EΔM-tM2e (EtM2e), H5_05_ (Influenza A/Hanoi/30408/2005 (H5N1), NCBI accession no. AB239125.1) (21) or H5_22_ (Influenza A/Red-Tailed Hawk/ON/FAV-0473-4-2022 (H5N1), EPI_ISL_17394087) (22) were cloned and inserted together with EΔM-tM2e into a VSVΔG vector at the position of the VSV-G gene using a previously described cloning strategy (23), and the resulting vaccine candidates were named V-EtM2e/H5_05_ and V-EtM2e/H5_22_.

**Fig 1:**
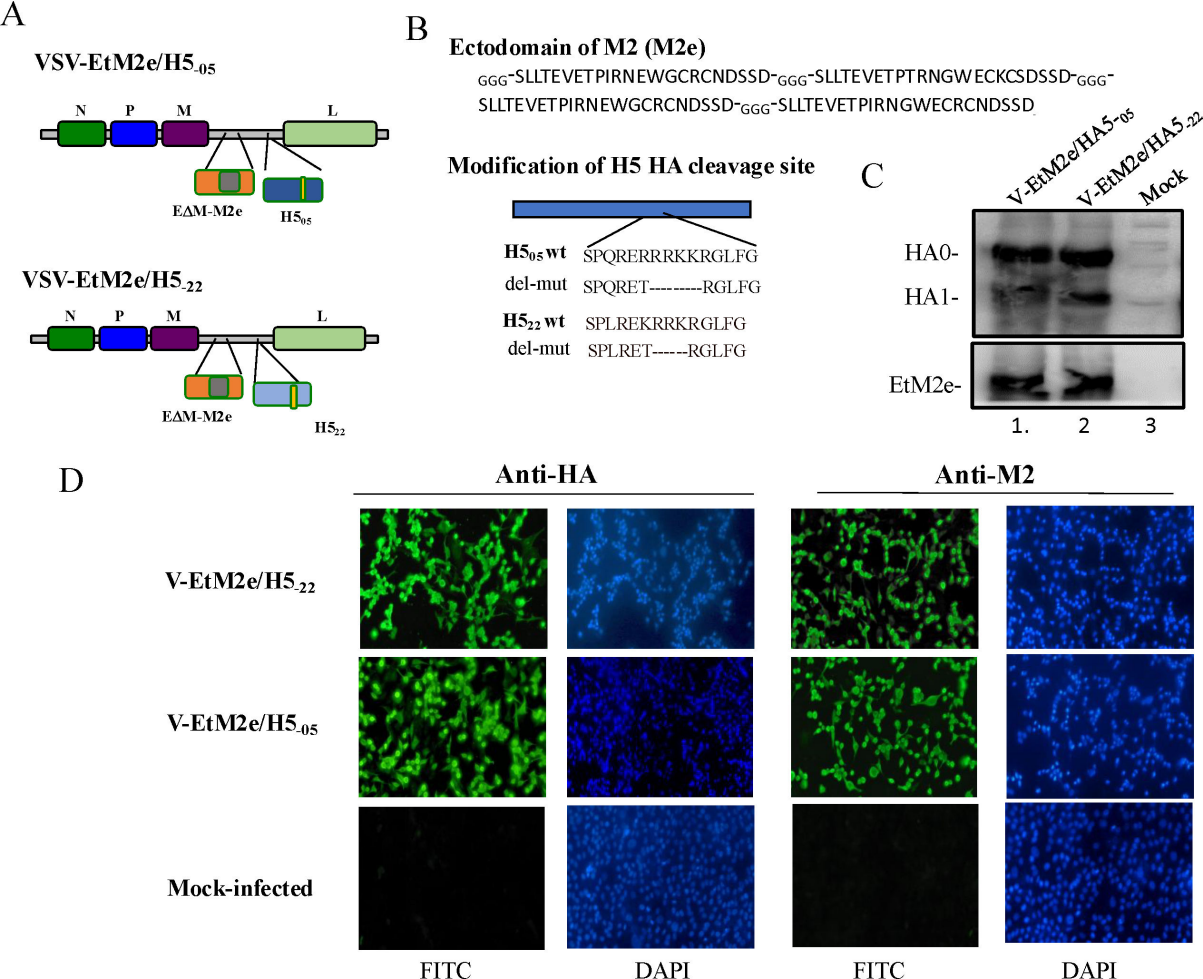
Construction and expression of V-EtM2e/H5_05_ and V-EtM2e/H5_22_ multivalent vaccines. (**A**) Schematic diagram of the EΔM-tM2e, H5_05_, or H5_22_ immunogens present in the VSV vaccines. (**B**) tM2e (top panel): The tM2e that was comprised of four copies of the conserved influenza M2 ectodomain (24 aa) polypeptide (tM2e), two copies from human flu strains, one from swine flu strain, and one from avian flu strain. Bottom panel: In both H5_05_ and H5_22_ open reading frames, a deletion was made to convert the furin-dependent cleavage site to a trypsin-dependent activation site. (**C**) VeroE6 cells infected with V-EtM2e/H5_05_ or V-EtM2e/H5_22_ were lysed and processed with SDS-PAGE, followed by WB with a rabbit anti-HA antibody (top panel), a mouse antibody against influenza M2e (bottom panel). (**D**) Representative immunoﬂuorescence images of Vero E6 cells infected with V-EtM2e/H5_22_ (top panel), V-EtM2e/H5_05_ (middle panel), or mock-infected (bottom panel). Infected cells were incubated with anti-HA antibody or anti-M2e antibody, followed by corresponding FITC-conjugated secondary antibodies. Cells were viewed under a computerized Axiovert 200 inverted fluorescence microscopy.

The pCAGGS-DNAs encoding HA(H5_05_), neuraminidase (NA) (NCBI accession no. AB239126.1), and M2_05_ (A/Hanoi/30408/2005) were previously described (24). cDNA encoding M2e from H7N9 (human and avian), H5 (avian), H1N1, H3N2, and influenza B was generated by two-step PCR and subsequently cloned and inserted into the pCAGGS-M2 expressor. The codon-optimized gene encoding HA(H5_22_) was synthesized by GenScript Biotech, Inc., and cloned and inserted into the pCAGGS vector. The protease cleavage site of H5_05_ or H5_22_ was modified by a two-step PCR technique to delete five or four basic amino acids (RRRKK for H5_05;_ KRRK for H5_22_) and add a threonine residue proximal to the cleavage site of the protein (Fig. 1B), as previously described (24). The lentiviral vector encoding the luciferase gene was previously described (25).

### Cells, antibodies, recombinant proteins, and viruses

The HEK293T, VeroE6, and BHK-T7 cell lines were cultured in DMEM. The ADCC bioassay effector Jurkat cell line (BPS Bioscience, Cat# 79733) was cultured in RPMI-1640 medium (ATCC modification). Madin-Darby Canine Kidney (MDCK) cells obtained from the American Type Culture Collection (ATCC) were cultured in minimal essential medium (MEM) (Gibco). The above mediums were supplemented with 5% or 10% fetal bovine serum and 1% penicillin and streptomycin (P/S; 100 units/mL and 100 µg/mL, respectively; Gibco cat# 15140-122) for maintenance. The antibodies used in this study included a rabbit polyclonal antibody against HA (Sino Biological, Cat# 11700-T54), a mouse monoclonal anti-influenza A virus M2 antibody (Santa Cruz Biotech, Cat# 14C2: sc-32238). The secondary antibodies used were anti-mouse IgG-HRP (GE Healthcare, Cat# NA931), goat anti-mouse-IgG (H + L)-HRP, goat anti-mouse-IgG2a-HRP, and goat anti-mouse-IgG1-HRP (Southern Biotech, Cat# 1037-05, 1081-05, or 1071-05). The recombinant proteins included influenza A H5N1 HA (A Cambodia/NPH230032/2023, Sino Biological, Cat# 409450-V08B), H5N1 HA (A/bald eagle/Florida/W22-134-OP/2022, BEI Resources, Cat# NR-59424), H5N2 HA (A/snow goose/Missouri/CC15-84A/2015, BEI Resources, Cat# NR-50651), H7N9 HA (A/Human/02,285/2017, BEI Resources, Cat# NR-51195), H3N2 HA (A/Missouri/09/2014, BEI Resources, Cat# 40494-V08B), and H1N1 HA (A/Puerto Rico/8/34, Sino Biological, Cat# 11684-V08H).

The replication-competent recombinant vesicular stomatitis virus (rVSV)-based influenza vaccines V-EtM2e/H5_05_ and V-EtM2e/H5_22_ were rescued in 293T/Vero E6 cells, propagated, and titrated as described previously (23). The influenza A H5N1 virus A/BC/PHL_2032/2024 (GISAID # EPI_ISL_19548836) [A/BC(H5N1)] (26) was used to infect mice in the challenge experiments at the NML, Winnipeg.

### Western blot and immunofluorescence assays

To detect the expression of different viral proteins, Vero E6 cells were infected with V-EtM2e/H5_05_ or V-EtM2e/H5_22_ for 48 h, and the infected cells were lysed 30 min on ice with RIPA buffer (containing 50 mM Tris-HCl, pH 7.4, 150 mM NaCl, 1% NP-40, 0.5% sodium deoxycholate, 0.1% SDS, and 5 mM EDTA) supplemented with 1× protease inhibitor cocktail. The cell lysates were mixed with loading buffer containing β-mercaptoethanol (2-ME) and run through a 10% SDS‒PAGE denaturing gel, followed by immunoblotting with various antibodies, as indicated. The protein bands were visualized using an enhanced chemiluminescence kit (Perkin Elmer Life Sciences).

To detect the binding ability of anti-M2 antibody in the vaccinated mouse sera, 293T cells were transfected with plasmids encoding M2 from different strains for 48 h. The cell lysates were then run on a 12% SDS‒PAGE gel, followed by immunoblotting with a V-EtM2e/H5_22-_immunized mouse serum pool.

For the indirect immunofluorescence assay, Vero E6 cells grown on glass coverslips (12 mm^2^) in 24-well plates were infected with V-EtM2e/H5_05_ or V-EtM2e/H5_22_ for 24 h. After infection, the cells on the coverslip were fixed with 4% paraformaldehyde for 15 min and permeabilized with 0.2% Triton X-100 in PBS. The cells were then incubated with primary antibodies specific for M2e or HA, followed by the corresponding secondary FITC-conjugated antibodies. The cells were finally viewed under a computerized Axiovert 200 inverted fluorescence microscope.

### Mouse immunization and viral challenge

For the immunization experiments, female BALB/c mice aged 6 to 8 weeks (five per group) were immunized IM (1 × 10^7^ TCID50) with the vaccine candidates (V-EtM2e/H5_05_ or V-EtM2e/H5_22_) and boosted with the same dose of each vaccine on day 14. At 28 days post-immunization, the mice were sacrificed, and serum samples were collected for various analyses.

For virus challenge and sample collection**,** 80 BALB/c mice were separated into 4 groups (each group has 10 male and 10 female mice). Two groups were intranasally (IN) vaccinated with 1 × 10^5^ TCID_50_ in 50 µL of V-EtM2e/H5_22_ vaccine (IN vaccination group) or 1 × 10^5^ TCID_50_ in 50 µL of VSV-EBOVGP (IN control group), and the other two groups were intramuscularly (IM) vaccinated either with a total of 1 × 10^7^ TCID_50_ of the V-EtM2e/H5_22_ vaccine delivered in two 50 µL injections into each hind leg (IM vaccination group) or injected in the same manner with 1 × 10^7^ TCID_50_ of VSV/EBOVGP (IM control group). During IN vaccinations, the mice were placed under isoflurane anesthesia and then intranasal vaccination. All the animals were boosted at 21 days after vaccination (dpv) using the same method.

Following serum collection at 41 dpv, the mice were transferred to the containment level 4 (CL4) facility at the National Microbiology Laboratory (Winnipeg, MB, Canada). Mice were challenged intranasally (IN) on day 42 under isoflurane anesthesia with 10× LD_50_ (10 PFU) of a highly pathogenic H5N1 strain (A/BC/PHL_2032/2024) in a total volume of 50 µL (25 µL per naris). The animals were weighed and monitored daily and euthanized if they were assigned a preapproved clinical score. Criteria scored included coat condition, activity, posture, respiration and breathing, mobility, and responsiveness, weight loss, and appearance of neurological signs of CNS infection including hindlimb paralysis, ataxia and loss of righting reflex. Weight loss greater than 25% or any signs of CNS infection resulted in immediate euthanasia. Half of the animals were necropsied at 4 days post-infection (dpi), and serum, lung, heart, kidney, and spleen samples were collected in 2 mL cryovials. The tissue samples were weighed and flash frozen at −80°C for further downstream analysis. All other mice were monitored until 18 dpi.

### Measurement of viral burden in the tissues

To measure the viral titers in the tissues that were collected at 4 dpi, a median tissue culture infectious dose (TCID_50_) assay was performed. MDCK cells were seeded into 96-well tissue culture plates as described previously (23) and brought into CL4 for the addition of tissue homogenate dilutions. Tissues previously collected from euthanized animals were thawed and homogenized with 5 mm sterile stainless steel beads using a Bead Ruptor Elite Tissue Homogenizer (Omni). The homogenates were clarified by centrifugation at 1,500 × *g* for 10 min, after which 10-fold dilutions of the tissue homogenates were made in viral growth media. Dilutions were added to MDCK cells in triplicate wells, and the cytopathic effect was assessed at 72 h post-infection. TCID_50_ values per gram of tissue were calculated using the Reed and Muench method.

### Measurement of vaccine-induced HA- and M2e-binding antibody titers by ELISA

To determine the HA- and M2e-specific antibody titers in immunized mouse serum samples, ELISA was performed as previously described (27). Briefly, 96-well plates were coated with recombinant HA or M2e (human, GenScript, Cat# RP20206) (1 µg/mL) proteins in coupling buffer (pH 9.6) at 4°C overnight. After blocking at 37°C with blocking buffer (1% BSA [wt/vol] in 1 × TBS) for 2 h, the ELISA plates were washed and incubated with 2× serially diluted mouse serum samples at 37°C for 1 h. Goat anti-mouse-IgG(H + L)-, anti-mouse-IgG2a-, or anti-mouse-IgG1-HRP (SouthernBiotech) was used to detect the antibodies binding to HA or M2e. After the samples were incubated with the substrate tetramethylbenzidine (TMB) solution and terminated with stop solution, the absorbance of each well at 450 nm (OD450) was measured. The endpoint titers of mouse serum samples were calculated using interpolation in GraphPad Prism 10.0, with a cutoff of 2.5 times the mean negative.

### HA pseudovirus preparation and neutralization assay

H5N1 HA-pseudotyped Luciferase (luc)-expressing viral particles (H5-Luc-pseudovirus) were produced by co-transfecting 293T cells with each of HA_05_ or HA_22_, and NA, or M2 expressing plasmids, the HIV packaging plasmid pCMVΔR8.2, and a lentiviral vector expressing Luciferase (pEF1-Luc/puro) (25). After 48 h of transfection, the supernatant containing H5-pseudoviruses will be filtered through a 0.45 μm filter and used for neutralizing experiments.

The neutralization assay was performed based on influenza H5-pseudovirus and 293T cells according to the previous method (24), with some modifications. Briefly, the H5-pseudovirus was pre-activated by trypsin treatment (100 μg/mL) for 30 min at 37°C before infection. Then, the trypsin-activated H5-pseudoviruses (about 10^4^ RLUs, 25 µL) were pre-incubated with 2× serially diluted inactivated mouse sera (starting from 1:20 dilution, 25 µL) in complete DMEM in a 96-well plate at 37°C for 1 h. Then, 293T cells (1.5 × 10^4^ cells/well, 50 µL) were added to each well and incubated at 37°C. After 48–72 h of infection, cells were lysed in luciferase lysis buffer (Promega; 30 μL/well), and the luciferase relative light unit (RLU) in the lysates was measured using Polerstar Optima microplate reader (BMG BioLabtech) according to the manufacturer’s instructions. The neutralizing titers or half-maximal inhibitory dilution (ID_50_) were defined as the reciprocal of the serum maximum dilution that reduced 50% in RLUs compared with no-serum controls. The ID_50_ was calculated by using sigmoid 4PL interpolation with GraphPad Prism 10.0.

### Antibody-dependent cellular cytotoxicity reporter assay

The ADCC reporter assay was performed by using the ADCC Bioassay effector Jurkat cell line, which expresses firefly luciferase under the control of NFAT (nuclear factor of activated T cells) response elements and mouse FcγRIV (BPS Bioscience). Briefly, 1 day before the assay, 293T cells (6 × 10^5^/well, 6-well plate) were transfected with each plasmid expressing HA_05_, HA_22_, or M2 which carrying the M2e of influenza H5N1 or H7N9, using Lipofectamine 2000 (Invitrogen, USA) to produce 293T-HA or 293T-M2 target cells. The next day, the immunized mouse sera were 3× serially diluted in Assay medium 2A (BPS Bioscience#79621) (starting from 1:30, 50 µL/well) in a 96-well plate, mixed with target cells (1 × 10^4^, 50 µL/well), and incubated at 37°C for 1 h. The Jurkat effector cells (6 × 10^4^, 50 µL/well) were added and cultured for overnight at 37°C with 5% CO2. Finally, 150 µL of one-step luciferase reagent (BPS Bioscience, Cat#60690) was added to each well, and the ADCC activities were determined by measuring the Luciferase from activated Jurkat cells using a Polerstar Optima microplate reader. The reading of the background well (only medium) was deducted. The no-serum controls were wells containing target cells and Jurkat cells. The fold of change (ADCC induction) was calculated as RLU (test − background)/RLU (no-serum control − background).

### NK cell isolation and antibody-dependent cellular cytotoxicity

Mouse spleens were harvested from Balb/c mice, were gently pressed through a 40 µm cell strainer (Sigma) using the flat end of a sterile 1 mL syringe plunger, and resuspended in RPMI 1640 cell medium. The cell suspension was then layered over Ficoll-Paque PLUS (GE Healthcare) and centrifuged at 1,800 RPM for 30 min at 4°C with centrifuge brake level of 0. Mononuclear cells were collected from the interface of the two layers and washed with RPMI 1640 and resuspended in the recommended isolation medium. Primary NK cells (CD3^-^DX5^+^) were enriched by EasySep mouse NK negative enrichment kit (Stemcell) or RWD mouse NK isolation kit (RWD). Then, NK cells were cultured in RPMI-1640 media (Gibco) supplied with 10% fetal bovine serum (FBS), 1% Penicillin-Streptomycin-Glutamine (PSG), 1.6 mM 2-mercaptoethanol (2-ME), and 1,000U/mL IL-2 for 3 days.

IL-2-activated NK cells (0.2–0.4 × 10^6^/sample) were co-cultured with 1:20 diluted serum from PBS or vaccine-treated mice and HEK293T cells with or without transfected expression of the target protein, at an E:T ratio of 1:1. Fluorochrome-conjugated anti-CD107a antibody was added at the start of stimulation with the concentration of 1 µL/sample, with the 2 μM protein transport inhibitor monensin (eBioscience) added after the first hour. Cells were incubated 4 h at 37°C, 5% CO₂, in parallel with positive controls (PMA/ionomycin Cell Stimulation Cocktail plus protein transport inhibitors, 1:500 diluted, eBioscience). Following incubation, cells were washed with FACS buffer and surface-stained using anti-CD3 and DX5 mAbs. Samples were acquired on a flow cytometer, and ADCC was detected as percentages of NK cells (CD3-DX5+) undergoing degranulation as marked by surface CD107a expression (28).

### Statistics

Statistical analysis of antibody levels was performed using an unpaired *t* test (with *P* ≥ 0.05 considered to indicate statistical significance) with GraphPad Prism 10.0 software. Two-way ANOVA with multiple comparison tests was used to analyze mouse survival data, and the Mantel‒Cox test was used to analyze tissue TCID data.

## RESULTS

### Construction and characterization of the VSV vaccines expressing the modified H5N1 HA protein and EboΔM-fused influenza M2 ectodomain

To generate a VSVΔG vector co-expressing the codon-optimized genes encoding EΔM-tM2e, with H5-2005 (H5_05_), or H5-2022 (H5_22_), each of these cDNAs was cloned and inserted into an rVSVΔG vector, as indicated in Fig. 1A, and named VSV-EtM2e/H5_05_ or VSV-EtM2e/H5_22_. EΔM-tM2e (EtM2e) was generated by using Ebola glycoprotein DC-targeting domain (EΔM) fusion protein technology (29) through the insertion of four copies of the influenza M2 ectodomain (24 aa) polypeptide (tM2e, composed of two copies from human flu strains, one from a swine flu strain and one from an avian flu strain) (Fig. 1B, top panel) into EΔM to increase the immunogenicity of tM2e (16). In both the H5_05_ and H5_22_ open reading frames, mutations were introduced at the furin-dependent cleavage site to convert the furin-dependent cleavage site to a trypsin-dependent activation site characteristic of low-pathogenicity influenza envelope glycoprotein (24, 30) (Fig. 1B, bottom panel).

After these VSV vectors were constructed, each vector was rescued to obtain the vaccine candidates, V-EtM2e/H5_05_ or V-EtM2e/H5_22_, in a mix of 293T/VeroE6 cells via reverse genetics technology (23, 31). Each vaccine candidate was subsequently tested for infection in Vero E6 cells. After 28 h of infection, the infected cells were collected to detect different immunogens in the cell lysates. High expression levels of H5_05_, H5_22_, and EtM2e in each corresponding type of VSV-infected cell were detected by western blotting (WB) using antibodies specific for HA and M2 (Fig. 1C). Immunofluorescence assays confirmed the presence of both the HA and M2 proteins in the infected cells (Fig. 1D). These results revealed the concomitant expression of two avian influenza membrane proteins, HA and M2, in cells infected with the VSV vector vaccine candidates.

### V-EtM2e/H5_05_ or V-EtM2e/H5_22_ induced robust anti-HA and anti-M2e IgG antibody responses in immunized mouse serum

**Fig 2:**
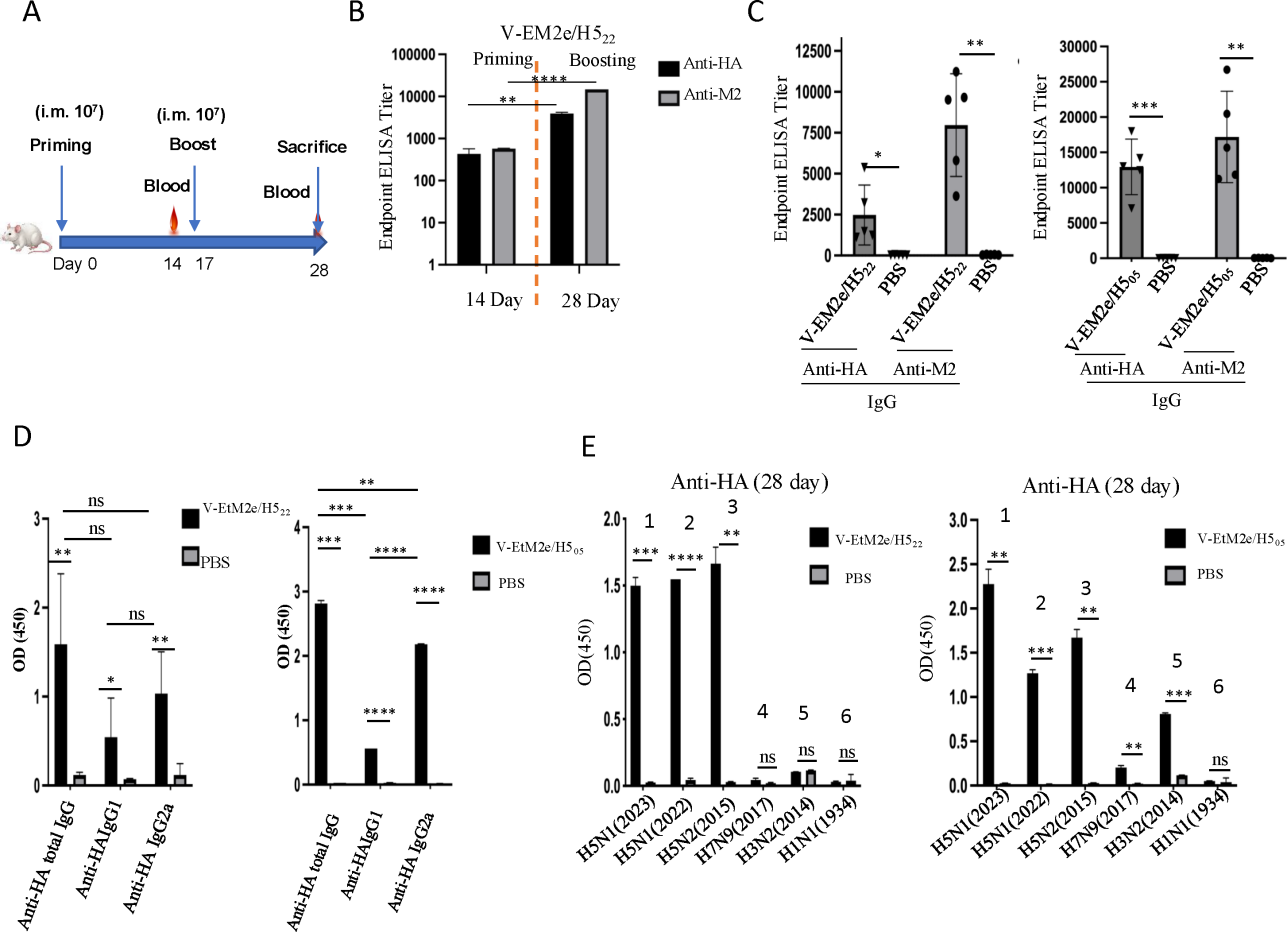
Anti-influenza HA and anti-M2e immune responses induced by rVSV vaccine candidates. (**A**) Schematic of the immunization of V-EtM2e/H5_05_ or V-EtM2e/H5_22_ via the intramuscular (IM) route in BALB/c mice. (**B**) The V-EtM2e/H5_22_ immunized mouse serum was pooled from individual mouse serum for each group collected on days 14 and 28 post-immunization and was measured for the endpoint titers of anti-HA and anti-M2 total IgG by ELISA. (**C**) The endpoint titers of anti-HA and anti-M2 total IgG in V-EtM2e/H5_22_ (left panel) or V-EtM2e/H5_05_ (right panel) immunized individual mouse serum (28 day). (**D**) The anti-HA total IgG, IgG1, and IgG2a antibody levels in V-EtM2e/H5_22_ (left panel) or V-EtM2e/H5_05_ (right panel) immunized mouse serum pools (1:200 dilution). (**E**) The levels of anti-HA antibody in V-EtM2e/H5_05_ (right panel) or V-EtM2e/H5_22_ (left panel) immunized mouse serum pool (1:200 dilution) by using ELISA with the recombinant HA proteins derived from different subtypes of influenza viruses, including H5N1, H5N2, H7N9, H3N2, and H1N1 by ELISA. Data represent mean ± SD from two independent experiments. Statistical significance was determined using an unpaired *t*-test. *, *P* < 0.05; **, *P* < 0.01; ***P < 0.001; ****, *P* < 0.0001.

In this study, the humoral immune responses induced by V-EtM2e/H5_05_ and V-EtM2e/H5_22_ were investigated by intramuscularly immunizing BALB/c mice with 1 × 10^7^ TCID_50_ of each vaccine product following a boost on day 17 (Fig. 2A). Sera from immunized mice were collected on days 14 and 28 postimmunization, and the levels of IgG antibodies specific to the HA of A/Cambodia/NPH230032/2023 (H5N1) or M2e (human) were determined by ELISA. On day 14, the anti-HA and anti-M2 IgG antibody endpoint titers in V-EtM2e/H5_22_**-**vaccinated mouse serum pool were only around 1:400 and 1:500 (Fig. 2B). As expected, on day 28 (11 days after boost), the serum anti-HA and anti-M2 IgG antibody endpoint titers were significantly increased (anti-HA, 1:3,900; anti-M2, 1:14,700) (Fig. 2B). We also evaluated the antibody levels in the serum of V-EtM2e/H5_22_- or V-EtM2e/H5_05_-vaccinated individual mouse (28 days), and the high levels of anti-HA and anti-M2 antibodies were detected in each immunized mouse (Fig. 2C). Anti-HA IgG subtypes (IgG2a and IgG1) in mouse serum were also quantified, and the results have shown that both V-EtM2e/H5_22_ and V-EtM2e/H5_05_ induced significantly higher levels of anti-HA IgG2a antibodies than anti-HA IgG1 antibodies (Fig. 2D). The above results indicate that these vaccine candidates are immunogenic and can induce efficient anti-HA and anti-M2e humoral immune responses.

### Specific recognition of different HA and M2 proteins from various influenza subtypes by vaccine-immunized sera

Next, we analyzed whether V-EtM2e/H5_22_ and V-EtM2e/H5_05_ vaccine-induced anti-HA antibodies could recognize recombinant HAs from different influenza subtypes, including H5 (recombinant HA synthesized through H5 genes isolated in different years), H1, H3, and H7 subtypes. The ELISA results revealed that both V-EtM2e/H5_22_ (Fig. 2E, left panel) and V-EtM2e/H5_05_ (Fig. 2E, right panel) immunization induced high levels of antibodies recognizing HA proteins from H5N1 (2022 and 2023) and H5N2 (2015) (Fig. 2E, bars 1–3). However, both types of vaccine-immunized sera, especially V-EtM2e/H5_22_-immunized sera, exhibited weak cross-reactive responses to H3 (2014) and/or H7 (2017) (Fig. 2E, bars 4 and 5) and very low binding to H1 (1934) (Fig. 2E, bar 6). Taken together, V-EtM2e/H5_05_ or V-EtM2e/H5_22_ immunization in mice induced a robust humoral response to HA from several H5N1 strains, as described here, but did not effectively recognize the HAs from the H1, H3, and H7 subtypes tested in this study.

### The anti-HA sera from V-EtM2e/H5_05_- or V-EtM2e/H5_22_-immunized mice induced high levels of specific neutralizing antibodies against H5-pseudovirus infection

**Fig 3:**
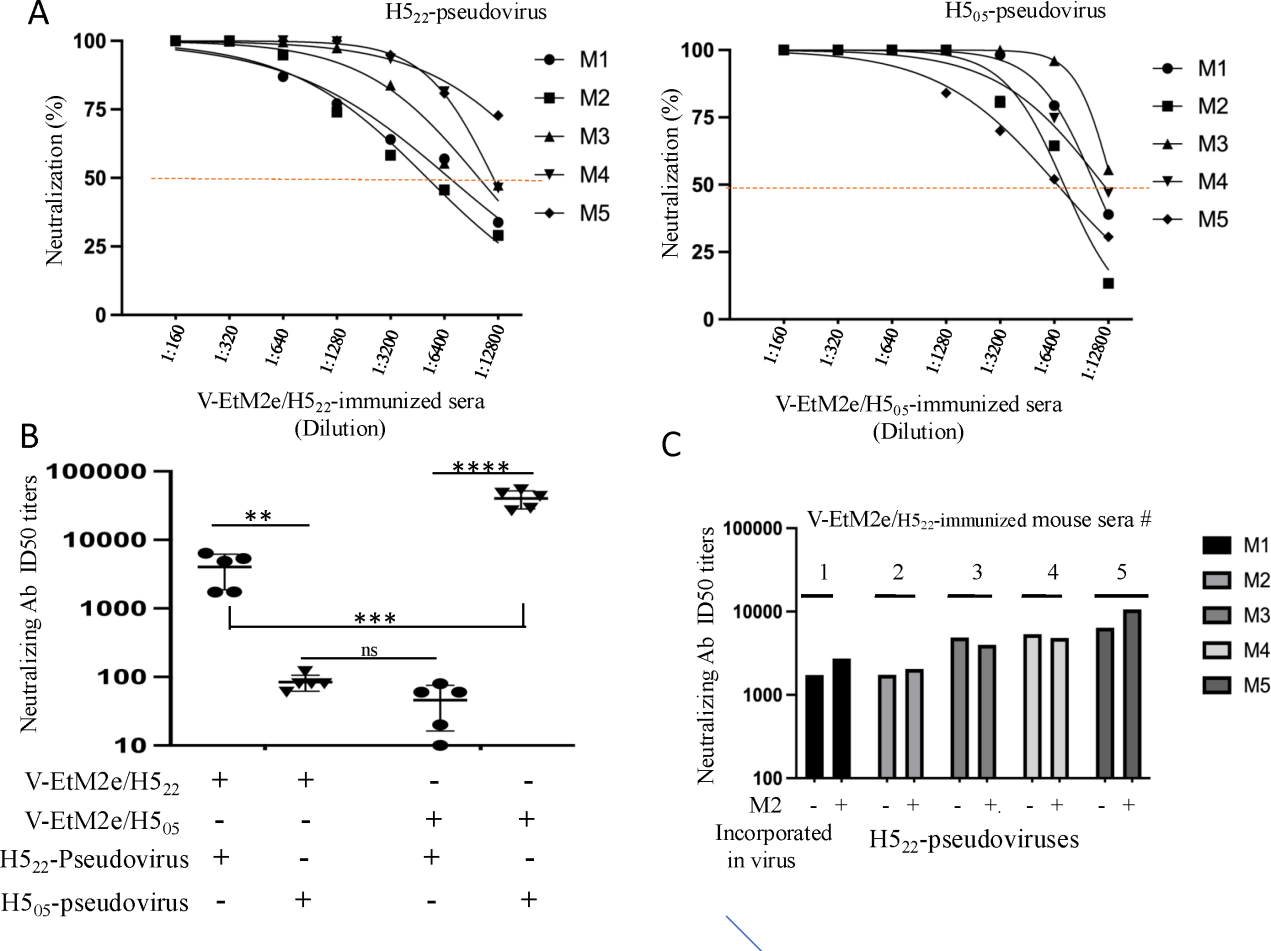
V-EtM2e/H5_05_ and V-EtM2e/H5_22_ vaccine candidates elicited neutralization antibodies. (**A**) Representative neutralization curves in individual immunized mouse serum against H5_22_- or H5_05_-Luc-pseudovirus. Normalized percentage neutralization values from V-EtM2e/H5_22_ (left) or V-EtM2e/H5_05_ (right) vaccinated mouse serum and plotted against the dilution factors. M1–M5: each of the individual immunized mice. Data represent the mean of triplicate wells from one representative experiment. (**B**) The ID50 titer of neutralizing antibody in each immunized mouse serum against H5_05_-Luc-pseudovirus or H5_22_-Luc-pseudovirus. Data represent mean ± SD of five mice and were obtained from over two independent experiments. Statistical significance was determined using an unpaired *t*-test. *, *P* < 0.05; **, *P* < 0.01; ***P < 0.001; ****, *P* < 0.0001. (**C**) The ID50 titer of neutralizing antibody in V-EtM2e/H5_22_ vaccine-immunized mouse serum pools against H5_22_-Luc-pseudovirus incorporated with or without M2 protein (as indicated). Data represent mean of triplicate wells from one representative experiment.

To evaluate whether immunization with V-EtM2e/H5_05_ or V-EtM2e/H5_22_ can induce protective immune responses against avian influenza H5-mediated viral infection, we tested neutralizing antibody (NAb) levels by using the H5-pseudotyped lentiviral virus (H5-PVLP) system in 293T cells, as described in the Materials and Methods. First, we analyzed whether each vaccine-immunized mouse serum sample could produce neutralizing antibodies against the corresponding H5-PVLP infection. The results clearly showed that all the V-EtM2e/H5_22_- and V-EtM2e/H5_05_-immunized mouse serum samples exhibited high neutralizing activity against the corresponding H5-PVLP infection (Fig. 3A). Conversely, the sera from V-EtM2e/H5_05_-immunized mice could not efficiently block H5_22_-PVLP infection, while the sera from V-EtM2e/H5_22_-immunized mice failed to effectively block H5_05_-PVLP infection (Fig. 3B). These findings substantially confirmed the strain specificity of NAbs induced by HA. Furthermore, we tested whether anti-M2e could enhance the anti-HA neutralization of M2^+^/H5_22_-PVLPs by detecting the neutralization antibody ID50 titers in V-EtM2e/H5_22_-immunized mouse serum against M2^+^ or M2^−^/H5_22_-PVLPs. All individual mouse sera inhibited M2^+^/H5_22_-PVLP infection and M2^−^/H5_22_-PVLP infection at similar levels (Fig. 3C), suggesting that the presence of anti-M2e antibodies in the serum did not affect the neutralizing activity mediated by anti-HA antibodies.

### Vaccination-induced broad ADCC activity against different influenza M2 proteins

**Fig 4:**
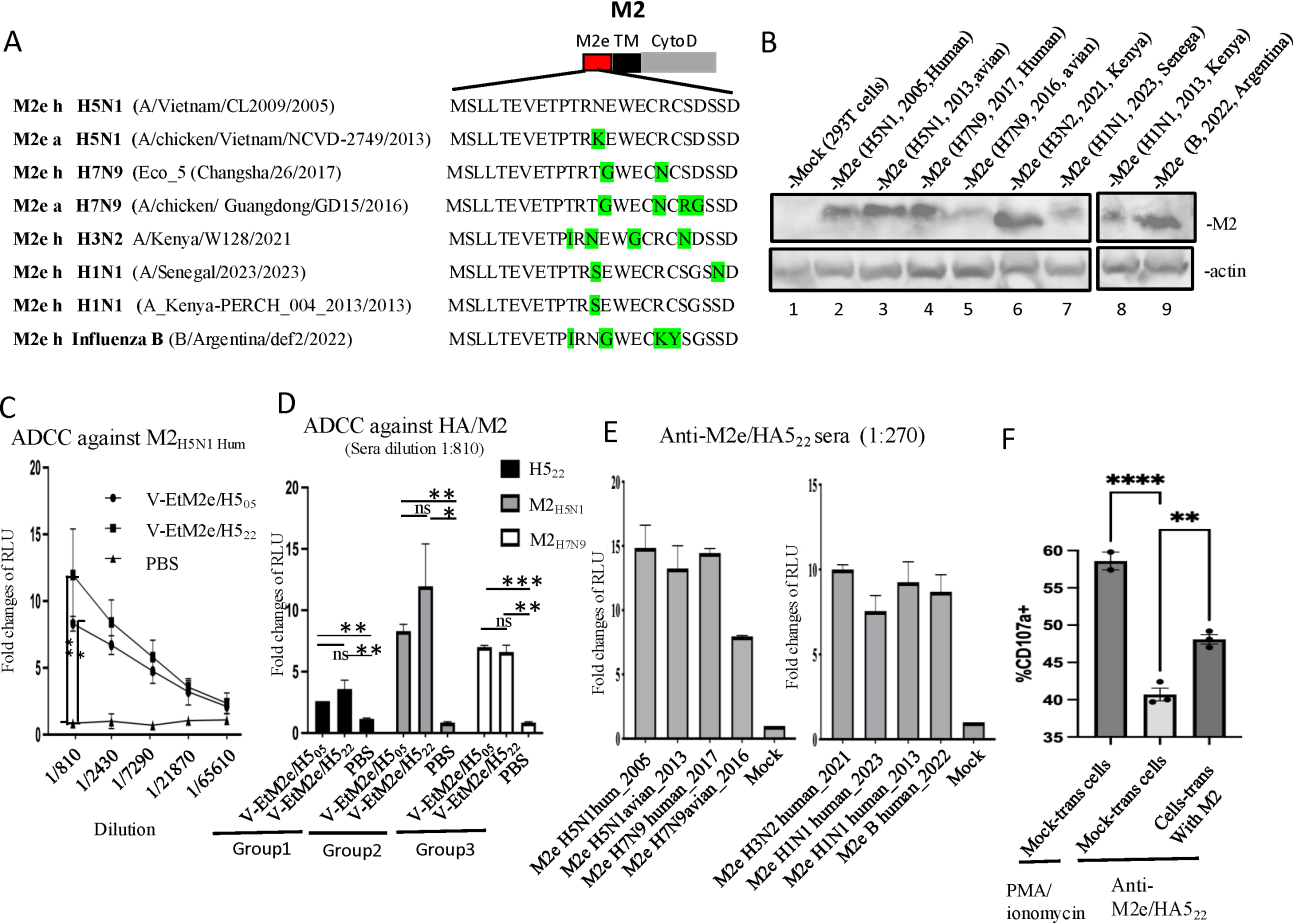
Vaccine immunization induced the ADCC activities against HA and M2e from a variety of influenza A subtypes. (**A**) Sequence alignments of the ectodomain of M2 proteins (M2e) from different subtypes of influenza A and B. Each M2e encoded gene sequence was fused in frame to the transmembrane domain and the cytoplasmic region of an H5N1 M2 expressing plasmid (on the top of right panel). The variable amino acids in M2e, which are different from H5N1 (human, 2005), are marked in green. (**B**) Each M2 plasmid encodes M2e from different subtypes of influenza (as indicated) and was transfected into 293T cells. After 48 h, the cells were lysed and run on a 12% SDS-PAGE gel, followed by immunoblotting with a pool of sera from V-EtM2e/H5_22_ immunized mice and an anti-actin antibody. (**C**) The anti-HA ADCC activity induced by V-EtM2e/H5_05_ or V-EtM2e/H5_22_ immunized mouse pooled sera (3× serial dilution) was detected by measuring the Luciferase activity from activated effector Jurkat cells and calculated as fold change against the no-serum control. (**D**) Comparison of the anti-H5_22_ (group1), -M2e_H5_ (group2), and -M2e_H7_ (group3)-ADCC activity levels mediated by pooled sera (1:810 dilution) from V-EtM2e/H5_05_- or V-EtM2e/H5_22_-immunized mice. (**E**) V-EtM2e/H5_22_-immunized serum (Anti-M2e/HA5_22_) (1:810 dilution) mediated broad ADCC activities against each M2e from variable subtypes of influenza A and B, which was expressed on the transfected target 293T cells (as indicated). (**F**) Isolated mouse NK cells were activated with IL-2 and co-cultured with 293T target cells either transfected to express the H5N1 M2 (*Trans*) or mock-transfected 293T cells in the presence of diluted Anti-M2e/HA5_22_. PMA/ionomycin stimulation was included as a positive control in the assay. NK cell degranulation was assessed by CD107a surface expression. Data were shown as mean ± SEM (experimental groups, *n* = 3; positive control, *n* = 2). Data represent mean ± SD. Statistical significance was determined using an unpaired *t*-test. *, *P* < 0.05; **, *P* < 0.01; ***P < 0.001; ****, *P* < 0.0001.

ADCC against influenza A virus infection has been shown to provide cross-strain protection and significantly contribute to viral clearance and the termination of viral infection (32, 33). We, therefore, evaluated whether V-EtM2e/H5_05_ or V-EtM2e/H5_22_ vaccination could induce ADCC activity against different subtypes of influenza M2e and H5 by using an ADCC reporter system, as described in the Materials and Methods (34, 35). To do so, we first constructed M2 plasmids expressing the gene encoding the M2 ectodomain from different strains, including H5N1 (human and avian), H7N9 (human and avian), H3N2 (2021), H1N1 (2023 and 2013), and influenza B (2022) (36), which was fused with the transmembrane domain and the cytoplasmic region of H5N1 (24) (Fig. 4A). To confirm the expression of different subtypes of M2e, each M2 expressor was transfected into 293T cells, after which the cells were lysed and processed for WB analyses by using V-EtM2e/H5_22_-induced anti-M2e antibodies in the serum of vaccinated mice. The results revealed that the serum of vaccinated mice can recognize the M2 ectodomain from a wide range of influenza A strains with variable binding affinities (Fig. 4B). The broad binding ability of the anti-M2e antibody from the vaccinated mice is expected since the ectodomain of M2 is highly conserved among viruses and the M2 ectodomain polypeptide that comprised of M2e from human, swine, and avian flu strains would further enhance the cross-strain binding ability (Fig. 1A and B). We next examined the ADCC activity mediated by immunized mouse serum against above influenza subtype M2e. The transfected 293T cells expressing M2 (H5N1 2005) were used as targeting cells, and the effector cell was ADCC Bioassay Jurkat cell line, which expresses mouse FcγRIV and firefly luciferase under the control of NFAT (nuclear factor of activated T cells) response elements. The data showed that both V-EtM2e/H5_05_- and V-EtM2e/H5_22_-immunized mouse serum pools induced strong ADCC activity against M2 in a dose-dependent manner (Fig. 4C). We then compared the anti-HA and anti-M2e ADCC activity levels of both immunized mouse serum pools (Fig. 4D). The results indicate that both V-EtM2e/H5_05_- and V-EtM2e/H5_22_-immunized mouse serum pools induced ADCC activity against H5_22_, with a two- to fourfold increase of luciferase activity at dilution of 1:810 (Fig. 4D, group 1). Interestingly, both immunized mouse serum pools induced significantly greater ADCC activity against M2_H5N1_- and M2_H7N9_-expressing cells (8- to 14-fold and 6- to 7-fold, respectively) (Fig. 4D, groups 2 and 3). Furthermore, we investigated the V-EtM2e/H5_22_-induced ADCC against M2e from different influenza viruses. The results revealed that M2 antibodies in V-EtM2e/H5_22_ immunized mouse serum pool can mediate ADCC activity against above the influenza subtypes tested in this study with 7- to 14-fold increase (Fig. 4E). Furthermore, we isolated the primary mouse NK cells and mixed them with V-EtM2e/H5_22_-immunized mouse sera (1:20 dilution) and the M2 (H5N1)-transfected 293T cells and incubated 4 h at 37°C, 5% CO₂. Then, cells were stained for NK identifying markers, and samples were acquired on a flow cytometer, and data were analyzed by gating on live cells. The results clearly showed that primary NK cells were activated in the presence of the immunized sera and M2_(H5N1)_-expressing 293T cells (Fig. 4F). Taken together, these findings demonstrate our vaccines' ability to induce functional antibodies that can mediate ADCC to influenza viruses.

### Immunization with V-EtM2e/H5_22_ via either the IN or the IM route efficiently protects mice from lethal H5N1 influenza virus infection

**Fig 5:**
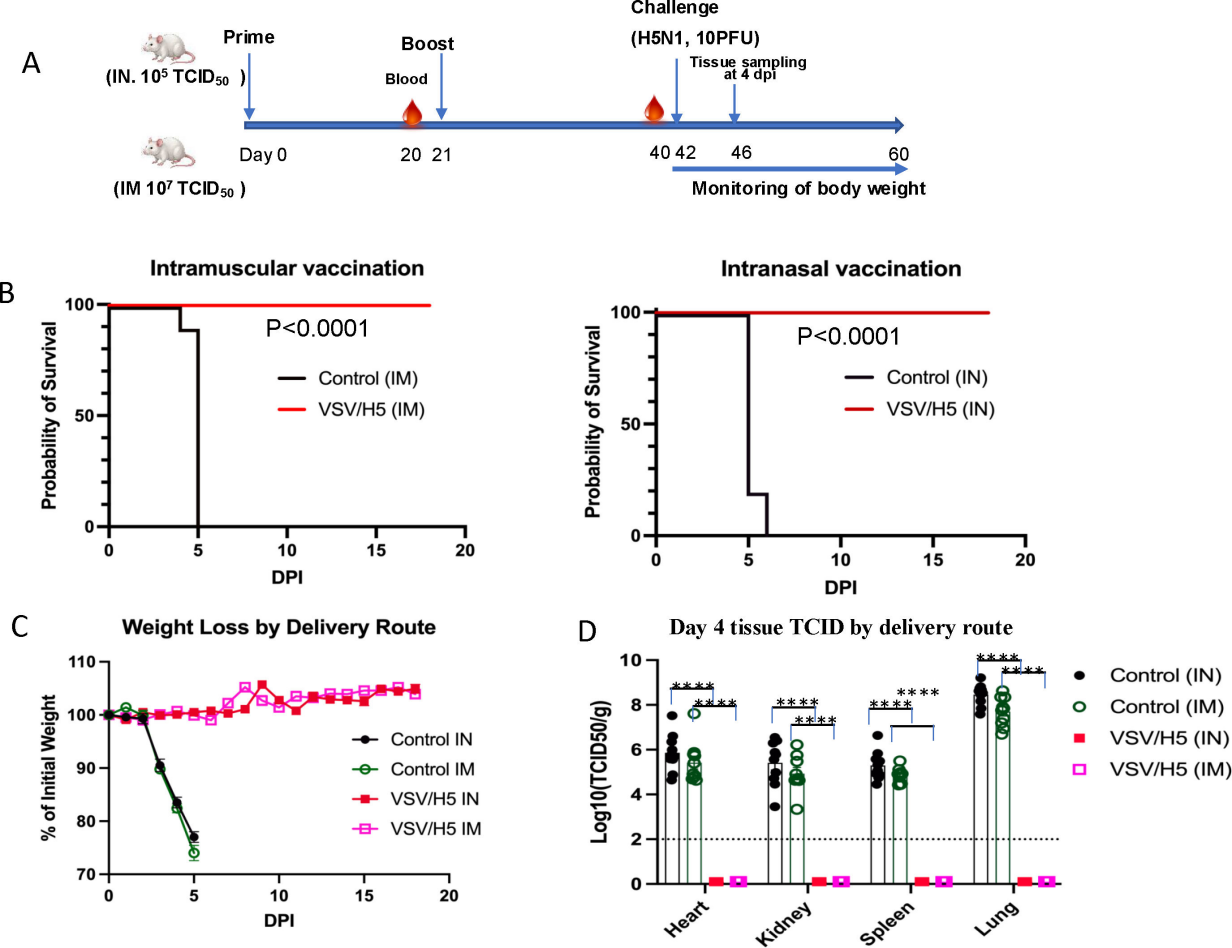
V-EtM2e/H5_22_ protected against lethal H5N1 influenza virus infection in mice. (**A**) Schematic of the VSV vaccine candidate immunization and H5N1 influenza challenge protocol used in the study. Briefly, BALB/c mice were administered with V-EtM2e/H5_22_ or VSV-EBOV-GP (control) via IM or IN at day 0 and day 21. Three weeks after the boost vaccination, the vaccinated mice were intranasally challenged with H5N1 virus (A/BC/PHL_2032/2024). Animals were weighed and monitored daily, half of the mice were euthanized and necropsied 4 days post-infection, and their serum, lung, heart, kidney, and spleen samples were collected for quantifying the infectious H5N1 virus in tissue homogenates. (**B, C**) Survival and weight loss in the vaccinated or the control mice following infection with the H5N1 virus. *n* = 20 at day 0 (dpi), through day 4; and *n* = 10 at day 5 through day 18 (dpi). (**D**) Viral burden in various tissues was detected by measuring the TCID_50_ values per gram of tissue in MDCK cells that were infected by tissue homogenates from challenged mice. *n* = 10 (from day 4 post-infection). Statistical significance was assessed by two-way ANOVA analysis with multiple comparisons (**A, B**) and the Mantel-Cox test (**D**). *, *P* < 0.05; **, *P* < 0.01; ***P < 0.001; ****, *P* < 0.0001.

To investigate the protective potential of the V-EtM2e/H5_22_ vaccine against H5N1 influenza virus infection, BALB/c mice (*n* = 40, 20 females and 20 males) were immunized with V-EtM2e/H5_22_ via the IM (1 × 10^7^ TCID_50_) or IN (1 × 10^5^ TCID_50_) route. As a control, VSV-EBOVGP was administered to mice (*n* = 40) at the same dose and via the same route. Two weeks after the boost vaccination, the mice were intranasally challenged with 10 × LD50 of the H5N1 virus (A/BC/PHL_2032/2024). Half of the mice (10 mice/group) were euthanized and necropsied at 4 days post-infection (dpi). The lung, heart, kidney, and spleen samples were collected to quantify the infectious H5N1 viral titer in the tissue homogenates (Fig. 5A). The remaining animals were weighed and monitored daily until 18 dpi. We found that both IM and IN administration of V-EtM2e/H5_22_ provided 100% protection against lethal H5N1 infection, whereas all the mice in the control groups died within 5–6 days (Fig. 5B and C). Importantly, no viral burden was detected in the heart, kidney, spleen, or lungs of IM- and IN-vaccinated mice after challenge with H5N1. In contrast, in VSV-EBOVGP vaccinated mice, H5N1 infection resulted in high H5N1 viral loads in different tissues, and the highest levels of viral loads were detected in the lungs (Fig. 5D). Data were also analyzed for differences between the responses of male and female mice to vaccination and virus challenge, and no significant difference of anti-HA antibody levels were found in male and female groups (Fig. S2A). Following challenge, female control group animals tended to exhibit clinical signs of illness, primarily weight loss, 1 day earlier than males and showed greater weight loss, although both males and females succumbed to the infection, or were euthanized, in the same time frame after infection (Fig. S2B). Consistent and significant differences in outcome after virus challenge were not noted in the vaccinated groups between males and females, with all animals in all groups maintaining or slightly gaining weight after virus challenge. In addition, there were no significant differences detected in antibody titers between males and females in either the IN or IM vaccination groups and lung virus titers in control animals at day 4 post-infection were not different between male and females (Fig. S2C). These results indicated that immunization with V-EtM2e/H5_22_ via either the IN or the IM route completely protected male and female mice from lethal H5N1 influenza virus challenge.

## DISCUSSION

A multivalent influenza vaccine capable of protecting against diverse influenza A strains, particularly highly pathogenic avian influenza (HPAI) strains, has been a central objective of influenza research for decades. In this study, we developed recombinant VSV-based vaccine candidates that coexpress HPAI H5 hemagglutinin (HA) and four copies of the influenza A matrix protein 2 ectodomain (M2e), which were fused to the dendritic cell-targeting domain of Ebola glycoprotein (EΔM) (Fig. 1A). Our findings illustrate that these candidates elicited strong immune responses against both HA and M2e, resulting in high neutralizing activity and robust antibody-dependent cellular cytotoxicity (ADCC) against different influenza subtypes. Most importantly, the immunized mice were fully protected against lethal challenge with a HPAI H5N1, highlighting the protective potential of this multivalent vaccine strategy.

Several recent studies have reported the development of universal influenza vaccines by genetic engineering of different viral antigenic domains to overcome the low efficacy of monomeric domain-based vaccines, including a chimeric HAs (cHAs) and the intact M2 mRNA vaccine (37), a combination of conserved influenza hemagglutinin stalk, neuraminidase, M2, and nucleoprotein combined mRNA vaccine (38), and a thermostable chimeric M2e and H3 hemagglutinin (HA) stalk protein vaccine (39). These studies have also demonstrated that immunization with these vaccines was able to confer protection against heterologous and/or heterosubtypic cross-group subtype virus infections. Our previous studies have demonstrated that the insertion of large heterologous polypeptides into the mucin-like domain (MLD) of the Ebola glycoprotein (EΔM) enables the efficient delivery of antigens to human macrophages and dendritic cells, inducing robust immune responses (29, 40). Building on this strategy, we incorporated four copies of M2e consensus sequences from different species of influenza strains (two from human strains, one from an avian strain, and one from a swine strain) into the EΔM scaffold to generate an EΔM-tM2e construct, as previously described (16) (Fig. 1A), which was designed to increase immune recognition of this conserved M2e antigen. In parallel, full-length cleavage-mutated H5_05_ or H5_22_ HA immunogens were inserted into the VSV vector, producing two vaccine candidates (V-EtM2e/H5_05_ and V-EtM2e/H5_22_) that simultaneously expressed both HA and M2e. WB and fluorescence analyses confirmed the efficient expression of H5_05_, H5_22_, and M2e in Vero-E6 cells infected with either VSV vaccine candidate (Fig. 1B and C). Consistently, animal immunization studies clearly revealed that these vaccines induced robust anti-HA and anti-M2e antibody levels (Fig. 2), demonstrating the high immunogenicity of the influenza antigens presented in this VSV-based vaccine platform.

We next examined the breadth of anti-HA responses induced by vaccination. Even though vaccinated mouse sera strongly recognized recombinant H5 proteins from the H5N1 strains isolated from 2015, 2022, and 2023, both sera bound weakly to H3 (2014) and H7 (2017) and could not bind H1 (1934) well (Fig. 2E), indicating that the HA-directed immune response is largely subtype restricted. It is expected since the HA proteins in different influenza A subtypes are highly diverged at the sequence level, with about 36% to 80% of amino acid identity (41). Among H5N1 influenza variants, the amino acid sequence identity of HA is 91%–94%. Interestingly, the anti-HA antibody induced by V-EtM2e/H5_05_ displayed a relatively higher affinity to H3N2 (2014) when compared with V-EtM2e/H5_22_ (Fig. 2E, left and right panels, Bar 5). By sequence alignment analysis, we found that the six amino acids at sites 88N/120L/149S/157S/197P are identical to H3N2 (A/Missouri/09/2014) in the HA head of H5N1(2005), while H5N1(2022) does not. It could be possible that these aa differences in the HA of H5N1(2005) might enhance the binding affinity to the HA of H3N2 (2014). This observation deserves further investigation.

Given that HA mediates viral attachment and entry, neutralizing antibodies play a central role in blocking early viral infection. Therefore, we have performed a luciferase-expressing H5-PVLP infection assay, as described in Materials and Methods, to evaluate the neutralizing antibody levels. The results revealed that the sera from V-EtM2e/H5_22_-immunized mice exhibited strong neutralization of H5_22_-PVLP infection but limited neutralizing activity against H5_05_-PVLPs, whereas the V-EtM2e/H5_05_-immunized sera showed the opposite pattern (Fig. 3A and B). Comparison of the H5_05_ and H5_22_ sequences revealed numerous mutations, particularly within the HA head domain, which governs receptor binding (Fig. S1). These differences likely explain the limited cross-neutralization. Above findings underscore the importance of accounting for variability in the HA head when HA-based vaccines are designed to optimize neutralizing capacity. Several vaccine strategies seek to redirect immune responses toward the conserved HA stalk, thereby improving the breadth of the vaccine and reducing the need for annual reformulation (42, 43). We observed that although neutralizing antibody titers against heterologous H5 were relatively low, partial inhibition at higher serum concentrations was observed (Fig. 3B), suggesting that enhancing stalk-directed immunogenicity with full-length HA vaccines remains important. Whether such variable neutralization is sufficient to confer *in vivo* protection warrants further investigation.

Another vital component of our vaccine design is the highly conserved ectodomain of the influenza matrix protein 2 (M2e), which is a promising universal influenza vaccine target because of its stability and limited sequence variation. Previous studies have shown that the ectodomain of the influenza virus M2 protein can mediate either antibody-dependent cellular cytotoxicity (ADCC) or antibody-dependent cellular phagocytosis (ADCP), which leads to the elimination of the influenza virus or the destruction of cells that are already infected (44, 45). In this study, four copies of influenza consensus M2e from human, swine, and avian flu strains were expressed, and we demonstrated that the anti-M2e immune responses induced by either V-EtM2e/H5_05_ and V-EtM2e/H5_22_ were not only able to recognize M2e derived from H5N1 but also bind to M2e derived from human and avian H7N9, H1N1, H3N2, and influenza B (Fig. 4B). Notably, vaccine-immunized sera were able to mediate strong ADCC activity against M2e derived from above influenza subtypes (Fig. 4C through E), suggesting potential broad protection of our vaccine candidates against different influenza A infections from various host ranges. Indeed, our previous study has demonstrated that immunization with EΔM-tM2e via the intramuscular or intranasal route provided full protection against lethal H1N1 and/or H3N2 influenza virus challenge (16, 25, 46). Our data suggest that both neutralizing and Fc-mediated ADCC may contribute to protection; however, their relative contributions were not directly evaluated in this study. Future studies, including passive transfer and FcγR-dependent models, will be required to dissect these mechanisms. A recent study has shown that the antibodies elicited by a chimeric hemagglutinin influenza virus vaccination confer FcγR-dependent protection (47). We will conduct the mechanistic studies to elucidate the relative contributions of vaccine-induced neutralizing and ADCC activity by using the FcγR-deficient mouse model. Also, further investigations will be conducted to test whether V-EtM2e/H5_22_ could mediate broad protection against infections by other human and avian influenza A viruses.

We finally evaluated the protective efficacy of V-EtM2e/H5_22_ against lethal H5N1 challenge in mice. Both IM and IN immunization conferred complete protection to male and female animals following exposure to a highly pathogenic H5N1 strain isolated in 2024. None of the immunized mice exhibited significant weight loss after infection, in stark contrast to the control animals (Fig. 5B and C). Notably, at 4 days post-challenge, the infectious H5N1 virus was not detected in multiple tissues, including the lungs, spleen, heart, and kidney of the V-EtM2e/H5_22_ immunized mice (Fig. 5D). These findings provide compelling evidence that V-EtM2e/H5_22_ (delivered via either the IM or IN route) can effectively prevent currently circulating H5N1 infection, clinical disease, and systemic viral replication in a mouse model. It should be noted that in this study, we only investigated the protective effect of V-EtM2e/H5_22_ against the H5N1 virus challenge. This is because the 2022 clade 2.3.4.4b H5N1 virus is currently widely distributed in wild birds, domestic poultry, and many mammalian species and is a significant zoonotic threat to humans, while the 2005 clade 1 viruses largely represent strains that are no longer in circulation. Furthermore, the 2022 viruses are considerably more virulent than the 2005 virus, causing death in mice at very low doses in as little as 4–5 days, therefore representing the most stringent test of vaccine efficacy available to us. Given the excellent protection provided by the vaccine against homologous virus challenge, further testing against heterologous H5 viruses will be done in a later study that we have planned. Together, the results of this study strongly support the further investigation of this vaccine platform for broader translational applications.

Since the mucosal membranes of the respiratory tract serve as the natural entry point for influenza viruses, these membranes represent the first line of host defense. Ideally, compared with IM vaccination, mucosal vaccination via the respiratory tract may elicit stronger local protective immune responses. The results of this study demonstrated that IN administration, even at 100-fold lower doses, achieved protection equal to that of IM administration (Fig. 5). This further supports the high efficacy of noninvasive mucosal delivery with significantly lower doses of the vaccine to block influenza infection. However, we still do not know if immunization via the IM route at a 100-fold lower dose would still be protective, which needs further investigation. It should also be noted that the trypsin-dependent sensitive site of H5 in our vaccine platform resulted in the expression of a low-pathogenic influenza envelope glycoprotein (Fig. 1B) (24, 30), which can improve the safety profile without impairing the immunogenicity of the vaccine candidate. Together, these studies provide evidence for the high efficacy and attenuated feature of this bivalent vaccine platform, which can be easily adapted to generate effective influenza vaccines against different strains of influenza virus.

Overall, the results of this study demonstrate that both V-EtM2e/H5_05_ and V-EtM2e/H5_22_ vaccines elicited strong influenza-specific immune responses, and the V-EtM2e/H5_22_ immunization has shown to provide full protection against lethal HPAI H5N1 challenge. By integrating HA, a potent but antigenically variable target, with M2e, a highly conserved epitope that mediates Fc-dependent effector functions, this platform achieves both subtype-specific neutralization and broader cross-strain protection. These findings highlight the promise of rVSV-based multivalent vaccines as a foundation for developing a universal influenza A vaccine. Future studies will be essential to evaluate the durability of protection, assess efficacy against additional influenza strains, and validate performance in nonhuman primate models.

## Data Availability

The data generated and/or analyzed during the current study are available from the corresponding authors.

## SUPPLEMENTAL MATERIAL

Fig. S1 jvi.00097-26-s0001.tiff Influenza HA amino acid sequence comparison of H505 (A/Hanoi/30408/2005) versus H522 (A/Red-Tailed Hawk/ON/FAV-0473-4-2022).

Fig. S2 jvi.00097-26-s0002.tiff Anti-HA antibody levels and viral burden in male and female groups.

ASM does not own the copyrights to Supplemental Material that may be linked to, or accessed through, an article. The authors have granted ASM a non-exclusive, world-wide license to publish the Supplemental Material files. Please contact the corresponding author directly for reuse.
