# Supplementary figures and images for: A VSV-vector vaccine simultaneously targeting H5N1 hemagglutinin and matrix protein 2 induces robust neutralizing and ADCC antibody responses and provides full protection against lethal H5N1 infection in a mouse model
Source: J Virol. 2026 Jun 16;100(7):e00097-26. doi: 10.1128/jvi.00097-26 (PMC13386899; doi:10.1128/jvi.00097-26)

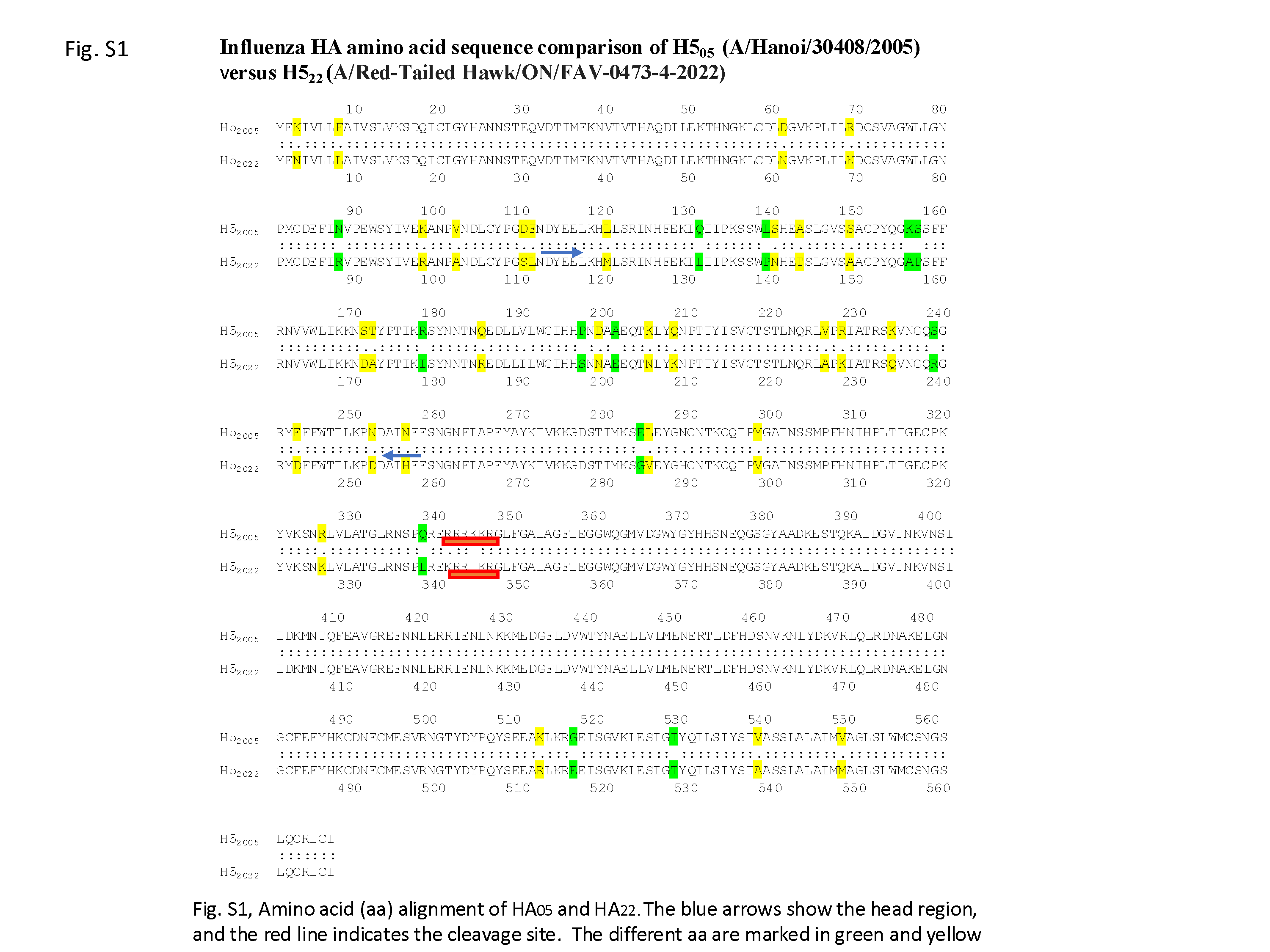

Supplement: Fig. S1 — Influenza HA amino acid sequence comparison of H505 (A/Hanoi/30408/2005) versus H522 (A/Red-Tailed Hawk/ON/FAV-0473-4-2022). [file jvi.00097-26-s0001.tiff]

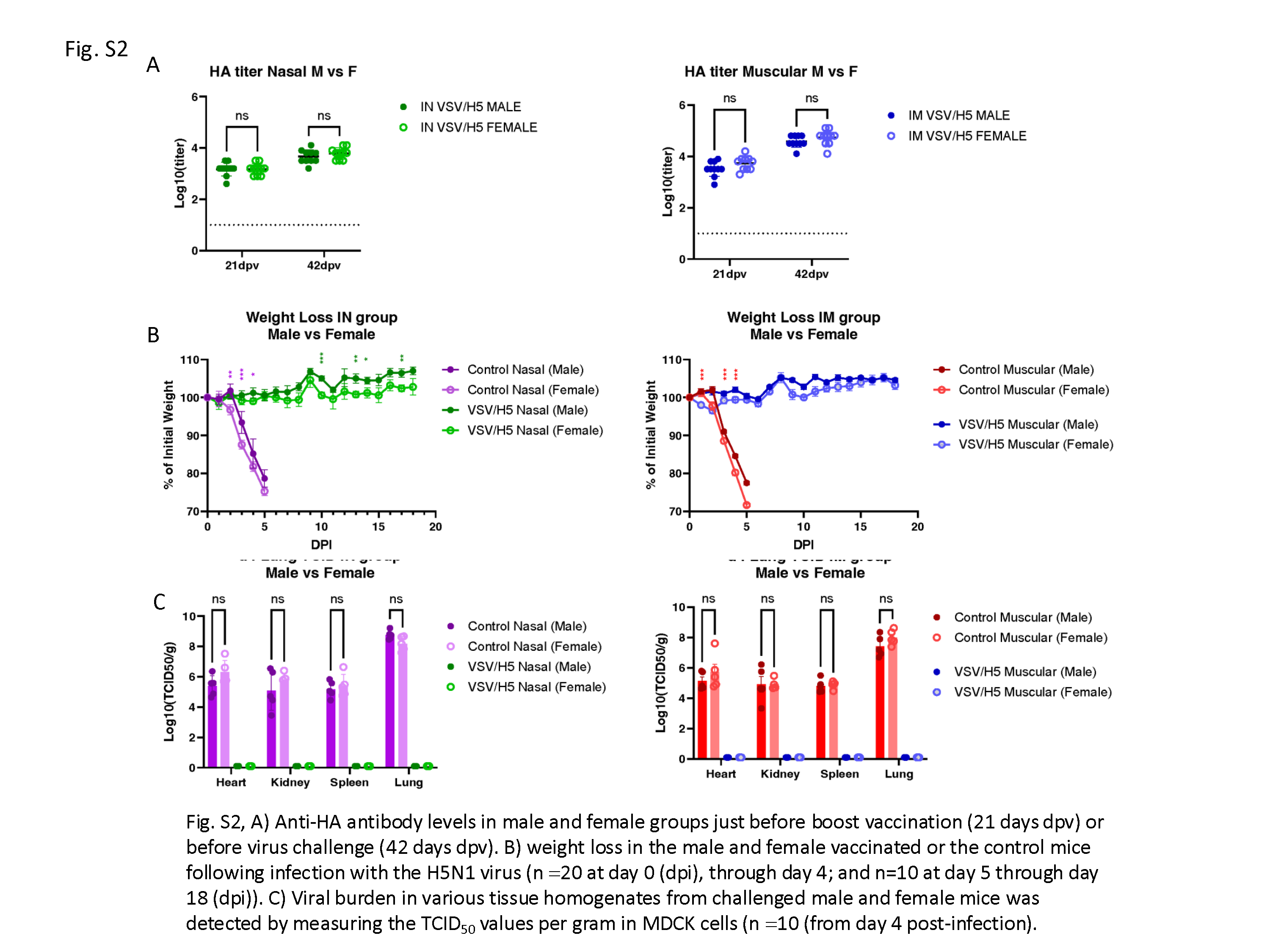

Supplement: Fig. S2 — Anti-HA antibody levels and viral burden in male and female groups. [file jvi.00097-26-s0002.tiff]
